# Isolation and Characterization of Cow-, Buffalo-, Sheep- and Goat-Milk-Derived Extracellular Vesicles

**DOI:** 10.3390/cells12202491

**Published:** 2023-10-20

**Authors:** Monisha Samuel, Rahul Sanwlani, Mohashin Pathan, Sushma Anand, Ella L. Johnston, Ching-Seng Ang, Maria Kaparakis-Liaskos, Suresh Mathivanan

**Affiliations:** 1 Department of Biochemistry, La Trobe Institute for Molecular Science, La Trobe University, Bundoora, VIC 3083, Australia19168073@students.latrobe.edu.au (R.S.); sushmaanand1616@gmail.com (S.A.); 2 Research Centre for Extracellular Vesicles, La Trobe University, Bundoora, VIC 3086, Australia; 3 Department of Microbiology, Anatomy, Physiology and Pharmacology, La Trobe University, Bundoora, VIC 3086, Australia; 4 Bio21 Institute, University of Melbourne, Victoria, VIC 2010, Australia

**Keywords:** extracellular vesicles, milk extracellular vesicles, cancer, milk EV proteome, bovine milk EVs

## Abstract

Milk is a complex biological fluid that has high-quality proteins including growth factors and also contains extracellular vesicles (EVs). EVs are a lipid bilayer containing vesicles that contain proteins, metabolites and nucleic acids. Several studies have proposed that EVs in cow milk can survive the gut and can illicit cross-species communication in the consuming host organism. In this study, we isolated and characterized extracellular vesicles from the raw milk of the four species of the Bovidae family, namely cow, sheep, goat and buffalo, that contribute 99% of the total milk consumed globally. A comparative proteomic analysis of these vesicles was performed to pinpoint their potential functional role in health and disease. Vesicles sourced from buffalo and cow milk were particularly enriched with proteins implicated in modulating the immune system. Furthermore, functional studies were performed to determine the anti-cancer effects of these vesicles. The data obtained revealed that buffalo-milk-derived EVs induced significantly higher cell death in colon cancer cells. Overall, the results from this study highlight the potent immunoregulatory and anti-cancer nature of EVs derived from the milk of Bovidae family members.

## 1. Introduction

Milk has constantly been consumed as a rich and dynamic source of nutrition [1]. It has evolved as a neonatal food that serves to ensure early development, growth and well-being in infants [2]. Its composition within mammalian species is indicative of the neonatal necessities of the offspring of that species. Milk provides the appropriate level of nutrients required by infants [3,4]. Furthermore, milk along with milk-derived products have always been considered as a rich repository of bioactive components that have been shown to produce positive health benefits [5,6,7]. It contains a vast array of bioactive compounds ranging from casein, enzymes, growth factors, cytokines, antimicrobials, microRNA and lipids [2,3,6]. In addition, milk also serves as a vector to transfer stem cells and commensal and probiotic bacteria to the recipient [8,9,10]. Currently, global milk production is mainly sourced from five animal species, namely cow, buffalo, goat, sheep and camel. According to statistics from the Food and Agriculture Organization of the United Nations (FAO), more than 99% of world milk production for consumption is sourced from four species of the Bovidae family, namely cow, buffalo, goat and sheep.

Living cells from all domains of life have been implicated to actively secrete membrane-bound vesicles in the extracellular space termed extracellular vesicles (EVs) [11,12]. In recent years, EVs have gained significant importance for their role in pathophysiology [13,14]. EVs have been isolated from a range of biofluids including blood [15], urine [16], cerebrospinal fluid [17], lymphatics [18], tears [19], saliva [20], nasal secretions [21], ascites [22], semen [23] and milk [24,25]. These vesicles serve as mediators of intercellular communication by playing an active role in the transfer of a highly diverse cargo between the cells [26]. The cargo transferred by EVs can be ubiquitous or cell-type-specific, eliciting a range of physiological responses in a living system [26,27]. Understanding the protein cargo of these EVs may provide an in-depth insight into their role in mediating inter-individual and cross-species communication and their impact on human health. Many studies have recently attempted to unravel the potential functions of these EVs isolated from human and bovine milk samples [25,28,29,30,31,32]. We have previously demonstrated that CoM (cow milk)-derived EVs are orally bioavailable and regulate cancer progression in vivo [28]. Furthermore, they sensitise aggressive, difficult-to-treat cancers including high-risk neuroblastoma and triple-negative breast cancer cells to chemotherapy [31,33]. Bovine-milk-derived EVs (MEVs) have also been defined as biocompatible and thus intrinsically suited to act as nanocarriers for drugs and molecules [32,34]. For instance, CoM-derived EVs have been engineered as a drug delivery vehicle. It has been shown that not only do these EVs efficiently package and deliver drugs, but they also possess anti-inflammatory and anti-cancer effects [32,34]. CoM-derived EVs have been shown to contain a large repertoire of immunomodulatory proteins that could play a critical role in regulating mammalian physiology [1]. They also contain microRNAs and mRNAs which have been speculated to play a vital role in the growth and modulation of the immune system [30]. We wanted to understand whether this anti-cancer effect is specific to EVs derived from CoM or is a conserved phenomenon across the Bovidae family.

In this study, we isolated and characterised EVs from the raw milk of the top four highly consumed species. Further to this, a comparative proteomic analysis was performed on the isolated vesicles and their potential functional role in health and disease was investigated. Additionally, functional studies were performed to evaluate the anti-cancer potential of MEVs from different animals from the Bovidae family.

## 2. Materials and Methods

### 2.1. Milk Samples

Animals were selected at random in this study. Pooled milk collected from Holstein cows was provided by Ellinbank Dairy Research Centre (Ellinbank, VIC, Australia). Pooled buffalo milk (BM) was obtained from Shaw river riverine buffalo farm (Yambuk, VIC, Australia). Pooled sheep milk (SM) and goat milk (GM) were kindly provided by Meredith dairy (Meredith, VIC, Australia) and CapriLac (Leeton, VIC, Australia), respectively. Prior to the study, all animals were healthy. Raw mature milk samples were collected from all the different species for comparative analysis. Milk samples were centrifuged immediately (within 3 h of procurement) at 2000× *g* for 15 min at 4 °C. The defatted samples were stored at −80 °C until further processing.

### 2.2. Isolation of EVs using Differential Centrifugation and Ultracentrifugation

Differential centrifugation and ultracentrifugation were used for the isolation of EVs from various raw milk samples, as previously described [1]. Briefly, the defatted milk samples were subjected to differential centrifugation (210 mL starting milk volume in SW-28 rotor, Beckman Coulter, 35 mL open top tubes, Life Sciences, Boston, MA, USA) starting at 5000× *g* for 30 min at 4 °C. The fat layer floating on top was removed carefully. After the removal of fat globules and milk-abundant proteins such as casein and cell debris, the skimmed milk samples were subjected to successive centrifugations at 4 °C for 1 h each at 12,000× *g*, 35,000× *g* and 70,000× *g*. Lastly, the supernatant obtained following these spins was centrifuged at 100,000× *g* using a SW-28 rotor (Beckman Coulter instruments, Waverley, VIC, Australia). The pellet obtained, containing enriched milk EVs, was resuspended in sterile, filtered PBS. A final washing step was performed using ultracentrifugation at 100,000× *g* for 1 h at 4 °C (Beckman Coulter: TLA-55 rotor). The resulting EV pellet obtained with this method was resuspended in the appropriate volume of PBS and stored at −80°C until further analysis.

### 2.3. Density Gradient Ultracentrifugation

Density gradient ultracentrifugation involved the use of a previously optimized method with modifications as needed [35]. Briefly, a discontinuous iodixanol gradient was prepared as follows: 40% *w*/*v*, 20% *w*/*v*, 10% *w*/*v* and 5% *w*/*v* solutions of iodixanol were prepared by diluting a stock solution of OptiPrep (60% *w*/*v* aqueous iodixanol from Sigma Life Sciences, Darmstadt, Germany) in 0.25 M sucrose/10 mM Tris, pH 7.5. The fractions were layered starting from the bottom with 3 mL each of 40%, 20% and 10%, reaching 2.5 mL 5% solution at the top. MEVs isolated from the milk of various animals using differential centrifugation were coupled with ultracentrifugation, resuspended in the OptiPrep solution and overlaid on top of the 5% layer. A control tube consisting of 3 mL of each 40%, 20%, 10% and 5% solutions was also prepared. All the tubes were simultaneously subjected to ultracentrifugation at 100,000× *g* for 18 h at 4 °C (Beckman Coulter: SW-28 rotor). Subsequently, 12 × 1 mL fractions were carefully removed and further processed to recover EVs. The fractions obtained were washed with 1 mL of 1 × PBS and subjected to ultracentrifugation at 100,000× *g* for 1 h at 4 °C (Beckman Coulter: TLA-55 rotor) twice to remove excessive reagents in the supernatant and obtain a relatively pure EV fraction resuspended in 100 µL of 1 × PBS before being stored at −80 °C.

### 2.4. Cell Culture

The human colorectal cancer cell line LIM1215 was kindly gifted by J Maraidason lab (Olivia Newton John Cancer Research Institute). Cells were cultured in RPMI 1640 medium (GIBCO™, Life Technologies, Carlsbad, CA, USA) supplemented with 10% (*v*/*v*) FCS (SAFC^®^ Bioscience, Sinking Spring, PA, USA) and 1% (*v*/*v*) 100 Units/mL of penicillin streptomycin (Gibco™, Life Technologies, Carlsbad, CA, USA). The cells were incubated at 37 °C with 5% CO_2_.

### 2.5. Western Blotting and Antibodies

SDS-PAGE was used to separate equal amounts of EV protein (quantified by Sypro^®^ Ruby staining). Proteins were transferred to the nitrocellulose membrane (Thermo Scientific, Waltham, MA, USA), blocked with 5% skim milk and probed with the following antibodies: TSG101 (BD Transduction Laboratories: catalogue number—612696) and Alix (Cell Signaling Technology, Danvers, MA, USA: catalogue number—2171S). Fluorescent conjugated rabbit and mouse secondary antibodies were used, and the protein bands were visualized using ODYSSEY CLx (LI-COR^®^).

### 2.6. Transmission Electron Microscopy (TEM)

EV samples (0.2 µg/µL) obtained from all four milk samples were examined in a JEM-2010 transmission electron microscope (JEOL, 80 kV, Akishima, Tokyo) or Tecnai TF30 transmission electron microscope (FEI, 300 kV, Lausanne, Switzerland), as described previously [35].

### 2.7. Nanoparticle Tracking Analysis (NTA)

NTA was performed on the various milk EV samples using ZetaView^®^ (Particle Metrix—ZetaView^®^, Inning am Ammersee, Germany) for direct quantification and determining size distribution. The absolute particle number was determined with ZetaView QUATT equipped with four lasers and ZetaView version 8.05.14SP7 software. The sample chamber was monitored using a 405 nm laser. MEV samples were diluted using Dulbecco’s PBS (DPBS) to obtain an average of 50–300 particles per field. The mean of three biological replicates was used to plot particle size distribution vs. particles per mL ± standard error of mean (SEM) using GraphPad Prism 8 Software.

### 2.8. FACS Cell Death Assay

An apoptosis assay was performed using a fluorescence-activated cell sorting (FACS)-based cell cycle analysis. Cells were seeded at a density of 25 × 10^3^ cells per well in a 24-well plate in 1000 µL RPMI 1640 culture medium. They were allowed to adhere for 2 days at 37 °C at 5% CO_2_. Cells were then treated with MEVs from various species at a concentration of 100 μg/mL and incubated for 72 h. The cells were then scraped and resuspended; 200 μL of cell suspension from each well was transferred into a 96-well plate and centrifuged at 300 g for 5 min. The supernatant was discarded, and the pellet was resuspended in 200 μL of PI-Hypotonic lysis buffer (0.1% (*w*/*v*) sodium citrate, 0.1% Triton X 100 (*w*/*v*), 50 μg/mL propidium iodide (Sigma Life Science^®^) in milliQ and incubated overnight at 4 °C. Samples were then subjected to FACS CANTO II (BD Biosciences, Franklin Lakes, NJ, USA) and analysed using FlowJo (TreeStar, Woodburn, OR, USA).

### 2.9. Gram Staining

Prior to the Gram staining, the milk samples were diluted in PBS. The diluted milk sample was dropped on top of a slide and spread to form a smear. The smear was heat fixed over a burner and allowed to air dry and cool. The smear was stained using crystal violet stain for a minute followed by washing with water to remove excess stain. The crystal violet stain was then fixed using iodine solution for 30 s and then excess iodine was removed by washing with water. The smear was then washed with ethanol. Lastly, the smear was counterstained with safranin for 60 s followed by washing with water to remove excess staining. The slides were then left to air dry prior to observing at 100× magnification (oil immersion) with a light microscope.

### 2.10. In-Gel Digestion

Equal amounts of proteins obtained from various MEV samples after OptiPrep density gradient centrifugation (fraction 7, 30 µg) were loaded onto precast NuPAGE^®^ 4–12% Bis-Tris gels in 1× MES SDS running buffer. Gels were run at a constant voltage of 150 V to resolve the proteins in EV samples. This was followed by staining the gels with Coomassie stain (Bio-Rad, Hercules, CA, USA) to visualize protein bands in individual lanes. Ten gel bands were excised from each sample lane in the gels and subjected to a previously optimized in-gel reduction procedure with alkylation and trypsinization [36]. Briefly, gel bands were reduced with 10 mM DTT (Bio-Rad), alkylated with 25 mM iodoacetamide (Sigma, Kanagawa, Japan) and digested overnight at 37 °C with 150 ng of sequencing grade trypsin (Promega, Madison, WI, USA). The following morning, the digested/tryptic peptides were extracted with 0.1% trifluoroacetic acid in 50% (*w*/*v*) acetonitrile.

### 2.11. LC-MS/MS

LC-MS/MS was performed using an LTQ Orbitrap Elite (Thermo Scientific, Waltham, MA, USA) equipped with a nanoESI interface in conjunction with an Ultimate 3000 RSLC nanoHPLC (Dionex Ultimate 3000, Sunnyvale, CA, USA). The nano-HPLC system was equipped with an Acclaim Pepmap nano-trap column (Dionex-C18, Sunnyvale, CA, USA, 100 Å, 75 µm × 2 cm) and an Acclaim Pepmap RSLC analytical column (Dionex-C18, Sunnyvale, CA, USA, 100 Å, 75 µm × 50 cm). Typically, for each LC-MS/MS experiment, the tryptic peptides (1 µL) were injected to the enrichment (trap) column at an isocratic flow of 5 µL/min of 3% *v*/*v* CH3CN containing 0.1% *v*/*v* formic acid for 6 min before the enrichment column was switched in line with the analytical column. The eluents used for the LC were 0.1% (*v*/*v*) formic acid with 5% (*v*/*v*) DMSO (solvent A) and 100% *v*/*v* CH3CN, 5% (*v*/*v*) DMSO and 0.l% (*v*/*v*) formic acid (solvent B). The flow gradient used was (i) 0–6 min, 3% B, (ii) 6–35 min, 3–23% B, (iii) 35–45 min, 23–40% B, (iv) 45–50 min, 40–80% B, (v) 50–55 min at 80% B, (vii) 55–56 min, 80–83% B and equilibration for 10 min before the next sample injection. Data-dependent mode with a nanoESI spray voltage of 1.9 kV was used to operate the LTQ Orbitrap Elite spectrometer, a capillary temperature of 250 °C was maintained and an S-lens RF value of 55% was used. All spectra were acquired in positive mode with full scan MS spectra scanning from *m*/*z* 300–1650 in the FT mode at 70,000 (QE) and 120,000 (Lumos, Waynesboro, VA, USA) resolution after accumulating to a target value of 1.0 × 10^6^. A lock mass of 401.9227 was used for both instruments. The top 20 most intense precursors were subjected to rapid-collision-induced dissociation (rCID) with a normalized collision energy of 30 and activation q of 0.25. Dynamic exclusion of 30 s was applied for repeated precursors.

### 2.12. Database Searching and Protein Identification

Peak lists were extracted from raw MS data into the Mascot Generic File Format (MGF) using MsConvert (version 3) with peak picking. The MGF files were then searched using X! Tandem (X! TANDEM Vengeance 2015.12.15.2). CoM, BM, GM and SM samples were searched against bovine, buffalo, goat and sheep protein databases downloaded from UniProt, respectively. BM, GM and SM samples were also searched against the bovine database as well. The following search parameters were used: fixed modification (carboamidomethylation of cysteine; +57 Da), variable modifications (oxidation of methionine; +16 Da), two missed tryptic cleavages, 20 ppm peptide mass tolerance and 0.2 Da fragment ion mass tolerance. Proteins were quantified using the Normalized Spectral Abundance Factor (NSAF) method [37].

### 2.13. Label-Free Spectral Counting

The relative protein abundance between the samples was obtained by estimating the ratio of normalized spectral counts as previously described [35]:

RSc for protein A = [(sY + c) (TX − sX + c)/(sX + c)(TY − sY + c)]

where s are the significant MS/MS spectra for protein A, T is the total number of significant MS/MS spectra in the sample, c is the correction factor set to 1.25, and X or Y are the exosome samples obtained from the milk of various animals (CoM, BM, GM and SM). When RSc is less than 1, the negative inverse RSc value was used.

### 2.14. Functional Enrichment Analysis

Functional enrichment analysis was performed using FunRich tool [38]. The heatmap, Venn diagram and gene ontology for the proteomic data was obtained from FunRich (version 3.1.4). Statistical analysis for gene set enrichment was performed using the inbuilt analysis tools in FunRich.

## 3. Results and Discussion

### 3.1. EV Markers Are Enriched in Fractions Corresponding to a Density of 1.08–1.22 g/mL

**Figure 1:**
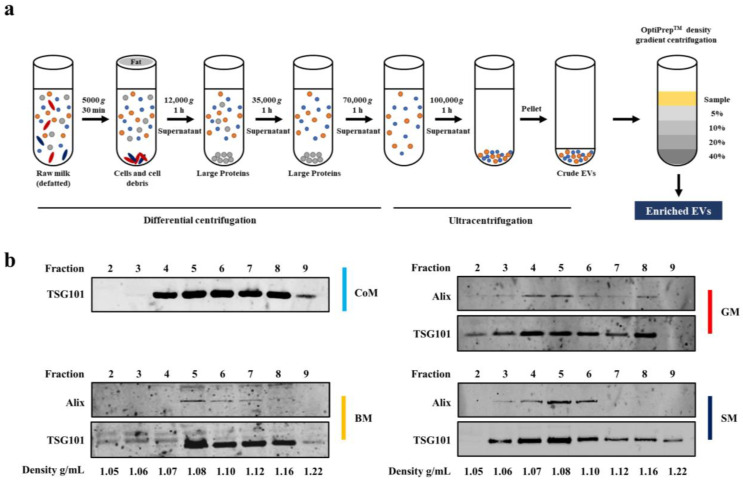
Isolation and characterization of MEVs from various species of the *bovidae* family. (**a**) Schematic representation of the differential centrifugation coupled with the ultracentrifugation-based method used to isolate and purify MEVs from various species. (**b**) Western blot analysis of various fractions of increased density obtained after OptiPrep density gradient centrifugation probed for EV-enriched proteins Alix and TSG101. Representative images of three biological replicates.

EVs were isolated from the mature milk samples of various species from the Bovidae family (cow milk—CoM, buffalo milk—BM, goat milk—GM and sheep milk—SM) using differential centrifugation coupled with ultracentrifugation [1]. The precise method used in this study for obtaining EVs is detailed in Figure 1a. Briefly, within 3 h of procuring milk samples, they were processed to remove intact cells and fat by subjecting them to low-speed centrifugation (2000× *g* for 15 min at 4 °C). The successive differential centrifugation of the defatted milk samples coupled with ultracentrifugation was performed to remove casein and other cellular debris and obtain crude EVs. The enriched EVs in the crude preparation were in the range of 10^11^–10^12^ particles/mL in a final volume of 750 µL from a starting volume of 210 mL of milk sample. A wash step was performed and the crude EVs obtained were further enriched using OptiPrep density gradient centrifugation (Figure 1a). To identify EV-enriched samples, the fractions obtained from density gradient centrifugation were subjected to Western blot analysis for detecting EV-enriched proteins Alix and TSG101 (Figure 1b) [39]. TSG101 was enriched in fractions 4–9 and 5–9 corresponding to the density of 1.08–1.22 g/mL in CoM and BM samples, respectively. Whereas, in GM and SM samples, EV-enriched proteins were detected in fractions 3–8 corresponding to the density of 1.06–1.16 g/mL. These observations are consistent with the findings in previous studies, indicating the buoyant density of EVs [1,24,28]. Alix was observed in fractions 4–7 in the BM, SM and GM samples (Figure 1b), but was not detected in any of the fractions in the CoM samples, as reported previously [1]. Fractions 5–7 from all the milk-derived EV samples were subsequently used for further analysis in this study.

### 3.2. Biophysical Analysis of MEVs

**Figure 2:**
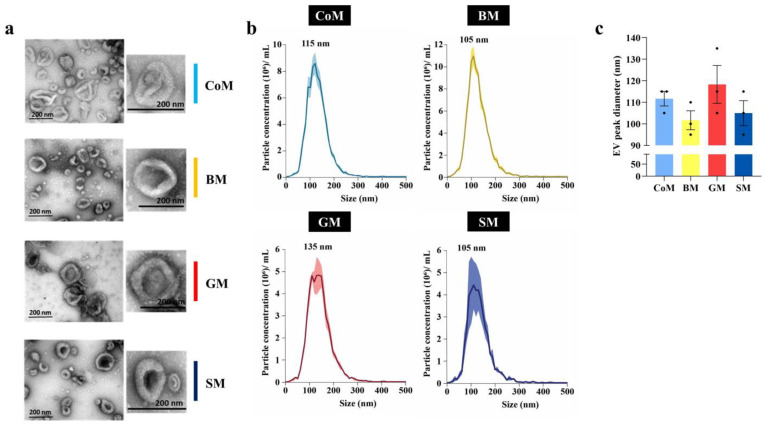
Biophysical characterization of EVs isolated from CoM, BM, GM and SM samples. (**a**) Representative TEM images of EVs isolated using OptiPrep density gradient centrifugation revealed vesicles with a morphology similar to EVs. (**b**) NTA revealed peak EV diameters at 115, 105, 135 and 105 nm for CoM, BM, GM, and SM samples, respectively. (**c**) Average peak EV diameter for individual biological replicates was compared for the samples. Data are representative of three biological replicates.

To further characterize the isolated EVs, they were subjected to biophysical analysis by performing TEM analysis and NTA. The vesicles detected in all four samples demonstrated properties that are characteristic of EVs. However, the vesicles detected were much larger, up to 370 nm, suggesting a heterogeneous mixed population of EVs. All the samples exhibited a rich abundance of a mixed population of EVs with predominantly intact vesicles consistent with typical EV-like morphology (Figure 2a). All four samples were analysed using NTA, allowing for the determination of their respective size distribution profiles (Figure 2b). Peak EV diameters (particle diameter for maximum particles in the sample) were detected to be 115, 105, 135 and 105 nm for the CoM, BM, GM and SM samples, respectively. The peak diameters of the EVs derived from the BM and SM sample were comparatively smaller (105 nm) than those from the CoM and GM samples. Moreover, the trend in peak EV diameter difference was maintained when the average of peak EV diameters for individual biological replicates was compared for the samples (Figure 2c). MEVs are highly heterogenous in nature and are secreted by a wide variety of cell types in the host ranging from mammary epithelial cells to immune cells and even the commensal or pathogenic bacterial strains present in milk [40]. In regard to this, several studies have recently indicated that EVs secreted by commensal and probiotic bacterial species could have a profound role in disease alleviation. Specifically, Gram-positive Lactobacillus species were observed to be anti-inflammatory in effect and had various health benefits ranging from alleviation of gut microbial dysbiosis, ulcerative colitis and inflammatory bowel disease to reduced proliferation and apoptosis induction in cancer cells [41,42,43,44,45]. In this study, the Gram staining of the milk samples obtained from these four species was performed to determine any variability among the milk obtained from various sources in terms of the bacterial populations present (Figure S1). It was observed that milk from all four bovine species was predominantly enriched with Gram-positive bacteria, both cocci (black dashed boxes) and bacilli (red arrows). Further, no Gram-negative bacterial populations were observed within any of the samples subject to Gram staining. These observations indicate the potential presence of Gram-positive bacterial EVs from species including Lactobacillus in the MEVs isolated from these four species and thus the associated benefits. However, further studies and analyses are needed to identify the bacterial strains present and the role of the EVs that they secrete in milk.

### 3.3. Proteomic Cargo Analysis of EVs Derived from the Milk of Bovidae Family Members

**Figure 3:**
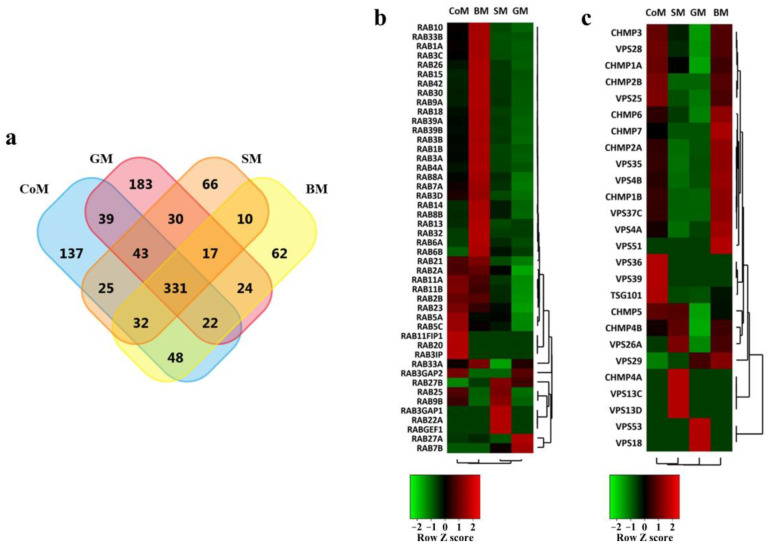
Proteomics analysis of EVs isolated from CoM, BM, GM and SM samples. (**a**) A four-way Venn diagram of proteins distributed between CoM, BM, GM and SM EVs revealing 331 proteins common to all four samples. (**b**) Heatmap showing enrichment of Rab proteins in CoM, BM, GM and SM samples. The scaled expression of each protein, denoted as the row *Z-score*, is plotted in the red–black–green colour scale. High expression levels are indicated in red and low expression levels are shown in green. The higher abundance of Rab family proteins was observed in the BM samples. (**c**) Heatmap depicting enrichment of EV proteins. EV-enriched protein TSG101 was comparatively highly enriched in CoM samples, whereas other EV proteins (VPS and CHMP) involved in the budding of these vesicles are more abundant in COMM and BM samples.

Next, to analyse the proteomic content of EVs derived from the CoM, BM, GM and SM samples, LC-MS/MS-based label-free quantitative proteomics was performed. Equal amounts of EV protein (30 µg) samples were separated using SDS-PAGE; gel bands were excised, reduced, alkylated and digested with trypsin. The extracted tryptic peptides were analysed on a LTQ Orbitrap Elite mass spectrometer. The resulting MS/MS spectrum was searched using X!Tandem against available sequence databases separately for all the species. Since BM, GM and SM are less studied as compared to CoM, number of proteins identified corresponding to those three species were very low in the databases in comparison to the latter. Thus, the Bos taurus RefSeq database was used to search against those species as well, as it would allow for the identification of either known proteins (i.e., those present in the database), or homologous proteins sharing identical peptides with the bovine database sequences. A total of 1069 proteins were identified across all four EV samples from various species (Figure 3a). Specifically, a total of 677, 546, 689 and 554 proteins were identified in EV samples derived from CoM, BM, GM and SM, respectively (Table S1). Further, 331 proteins were commonly identified in all four EV samples while 137, 62, 183 and 66 proteins were unique to CoM, BM, GM and SM samples, respectively. The number of proteins identified in BM and SM samples were comparatively lower than that in the other two species. This low number of proteins identified in the EVs of BM and SM samples could be imputed to the incomplete genomic database available and the lower number of studies conducted for these species.

### 3.4. EV Markers Are Enriched in Samples Derived from All Species

In order to assess the abundance of known proteins implicated in the biogenesis and trafficking of MEVs in all the species, the quantitative proteomic data were analysed. A heatmap was generated for endosomal sorting complexes required transport (ESCRT) components, Rab GTPases, vacuolar protein sorting (VPS) proteins and charged multivesicular body proteins (CHMPs), which were at least two-fold more abundant in all the samples. As depicted in Figure 3b, Rab GTPases that are related to endocytic and secretion pathways, as well as documented for their roles in both membrane transport and fusion, are present in all four samples [46]. Interestingly, the BM samples were comparatively richer in the presence of Rab family proteins as compared to the other species. Many of the other ESCRT component proteins, including CHMP and VPS family proteins, were also enriched in BM and CoM samples (Figure 3c). Classical EV-enriched protein TSG101 was observed to be found in CoM and BM samples in higher abundance (Figure 3c). Some other common proteins concomitant with milk, like Butyrophillin, Xanthine dehydrogenase, Ezrin, Adipophilin (Perilipin-2) and Lactadherin (MFG-E8), were also present in higher abundance in the EVs (Table 1). In total, 11 of the top 20 proteins identified in these EVs were common for all four species (Figure S2). The proteins that were uniquely identified in the EVs of specific species reflect the intrinsic characteristics of the milk fractions of these species. They may help us to better understand the differences in the physiology of mammary secretion among the studied animals.

### 3.5. Functional Analysis of Proteins Identified in the EVs

**Figure 4:**
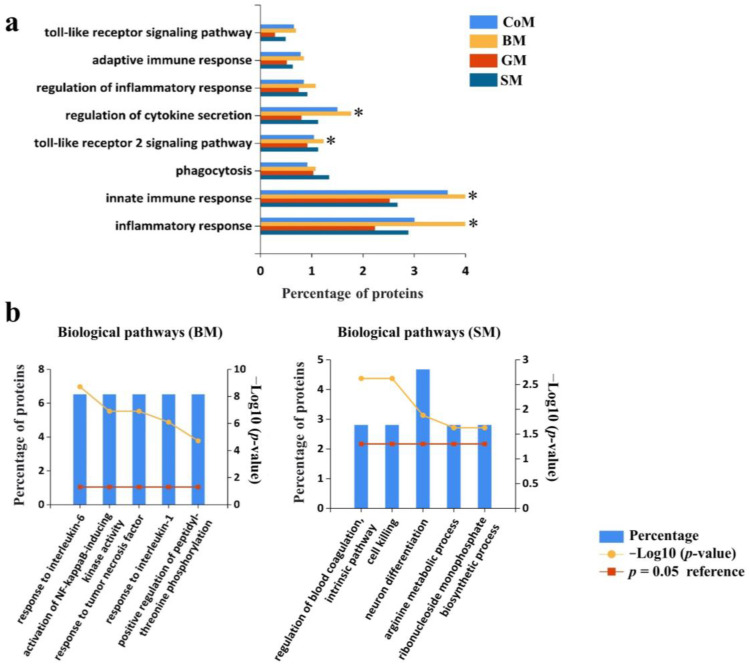
EVs from BM are enriched with proteins implicated in immune response. (**a**) Functional enrichment analysis using FunRich revealed that various proteins associated with innate immune response, inflammatory response and cytokine secretion were highly enriched in EVs isolated from BM. * Denotes *p* < 0.05. (**b**) Histogram representing top five biological pathways for proteins exclusively present in BM and SM samples.

To understand the potential physiological function of the EV proteins identified in various samples, we performed a functional analysis of all the two-fold more abundant proteins in the studied animals. As milk is known to play an extensive role in providing immunity to the neonate, all the proteins identified were classified into immune-related roles according to their annotated functions. Interestingly, it was observed that proteins associated with the innate immune system and eliciting inflammatory response were present in higher abundance in BM samples, comparatively (Figure 4a). Proteins regulating cytokine secretion, which has major immunomodulatory implications, were also enriched in BM, closely followed by CoM samples. Other innate sensing proteins like Toll-like receptors were also present in higher proportion in BM samples. Toll-like receptor-2 signalling proteins, which have been known as the central immunomodulator of PAMP-induced proinflammatory cytokine production, were also found to be significantly enriched in the BM EV samples (Figure 4a) [47,48,49]. Antimicrobial proteins including mucin and lactotransferrin were equally detected in higher abundance in all the samples. Mucins are important constituents of the extracellular matrix and are involved in various immune-related and cellular functions, whereas lactotransferrin has been known to provide protection against pathogenic infection [50,51,52]. We further analysed the biological pathways for proteins that were two-fold-enriched and exclusively present in CoM, SM, BM and GM samples, respectively. The top five biological pathways for CoM and GM samples reflected mostly intrinsic cellular processes like translation, myoblast differentiation and the regulation of mitochondrial depolarization (Figure S3). On the other hand, analysing SM samples showed pathways involved in blood coagulation, cell killing and neuronal differentiation (Figure 4b). Further, BM samples were most interesting as they mirrored pathways which responded to interleukins and tumour necrosis factor (TNF) as well as the activation of NF-κB kinase activity (Figure 4b). Interleukins are major players in systemic inflammation and are released in response to a stimulus, such as an infectious agent [53]. NF-κB family members regulate the transcription of cytokines and antimicrobial effectors. They also control cellular differentiation, survival and proliferation, thereby influencing innumerable facets of innate and adaptive immune responses [54].

### 3.6. BM-Derived EVs Induced Higher Cell Death as Compared to MEVs from Other Bovine Species

MEVs are known to potentially modulate immune cell function and affect the immune system [1,24]. Further, bovine milk EVs have been speculated to have a tumour targeting ability and to enhance the efficacy of chemotherapy drugs [31,32,33,34]. Thus, we determined the effect of these EVs in vitro on the cell cycle progression of colon cancer cells (LIM1215) using flow cytometric analysis to decipher any inherent anti-tumour activity. To verify this, cells were treated with MEVs from various species with a concentration of 100 μg/mL and incubated for 72 h. The concentration and time point for the treatment was determined by analysing our previous results where we observed a significant increase in cell death for the treatment of LIM1215 cells with CoM-derived EVs. Consistent with our previous results, we did observe an increase in cell death (% sub-G1) when cells were treated with BM- and CoM-derived EVs as compared to remaining untreated (UT) (Figure S4a). However, the cell cycle progression analysis showed that the effect of BM and CoM EVs was only restricted to the sub-G1 phase of the cells (Figure S4b). Interestingly, no significant difference in cell death was observed with GM and SM samples (Figure S4a). The results demonstrate that MEVs, specifically from CoM and BM, have direct anti-tumour activity and can induce cell death in cancer cells. Among all the various studied animals, BM-derived EVs showed the highest cell-death-inducing activity with a more than four-fold increase in cell death in LIM1215 cells.

## 4. Discussion

Milk is an exceptional source of both macro- and micro-nutrients and can therefore play a vital role in aiding individuals to meet their nutritional necessities [55,56,57]. Studies have shown that milk provides an expansive variety of biologically active compounds that aid in facilitating an infant’s growth and development. Further, milk components also serve to protect neonates and adults against diseases [2,3,5]. The role of dietary EVs from milk in facilitating species crosstalk is now being increasingly probed. Several studies so far have demonstrated the exemplary potential of these nanovesicles in driving physiological processes in the consuming organism [40]. Cow, buffalo, goat and sheep account for about 99% of global milk production [58]. However, studies so far have mostly focused on the role of cow milk and its constituents, leaving a knowledge gap as the nutritional and therapeutic value of EVs from the milk of other species remains largely unexplored [59]. Furthermore, most studies to date that have characterized milk EV cargo have largely explored the miRNA cargo with limited knowledge of protein and lipids in milk EVs, specially from minority species such as goat and sheep [60].

In the current study, milk EVs from four members of the bovidae family, cow, buffalo, goat and sheep, were isolated, characterized and their protein cargo was analysed using quantitative label-free proteomics. The isolated vesicles demonstrated biophysical characteristics typical of exosomes. For the first time, a comparative proteomic analysis on the EVs from various milk samples was performed. Using CoM as reference, comparisons of the proteomic content of the EVs were made among the species. We identified a variety of proteins like Rabs, CHMPS and VPS that are very commonly enriched in EVs and regulate the biogenesis and trafficking of these vesicles [11,26,27]. Additionally, the commonly reported abundant milk EV proteins such as butyrophillin, xanthine dehydrogenase, lactadherin and adipophilin were also identified [61,62,63]. The uptake of CoM-derived EVs by various cell types and their immunoregulatory potential has been previously demonstrated [1,30,40,64,65,66]. In the current study, a comparative functional enrichment analysis of the identified proteins revealed immune-related proteins to be found in higher abundance in BM-derived EVs as well as in CoM-derived EVs. These findings implicate a potential immunomodulatory role of these vesicles in the consuming organism.

Significant differences have been found in both macro- and micro-nutrient contents in milk sourced from various species [55,58]. Due to a lack of adequate studies on other species of the bovidae family, it has been difficult to compare the resulting effects of these variations in milk composition between species. Also, the highly abundant proteins mask the presence of unique ones which are less enriched and could play a substantial role in understanding the differences between the milk of these animals. We further performed a functional analysis by using an FACS cell death assay to determine the effect of these EVs on colon cancer cells. Interestingly, a four-fold increase in cell death was observed upon treatment of the tumour cells with BM-derived EVs. However, there was no significant difference in cell death observed upon treating the cells with EVs from SM and GM samples. This highlights the potent anti-tumour nature of BM-derived EVs by inducing higher cell death in cancer cells when compared to other MEVs. These results also clearly highlight the variation in the functional consequences of milk at the species level.

Overall, the results from this study help in augmenting our understanding of the EV proteome of vesicles from the top four highly consumed milk varieties and their likely physiological role. CoM proteins are one of the earliest and more comprehensively studied bioactive agents in milk [67]. However, recently, the nutritional value of BM has been shown to be higher than CoM owing to the higher amount of protein, fat, lactose, total solids and non-fat solids content, and the high buffer capacity [68]. Corroborating with this, the higher anti-cancer activity of BM-derived EVs could also be attributed to some of these factors. Nevertheless, further studies are essential to understand which components of these EVs are crucial in eliciting such responses. Also, studies are required to comprehend the type of cell death triggered by the BM-derived EVs. Numerous studies encompassing EVs from milk and other dietary sources have demonstrated their therapeutic significance in variety of diseases. However, there is a constant debate surrounding their oral bioavailability and function primarily due to lack of in vivo evidence explaining EV stability in the gut and their intestinal uptake to enter systemic circulation [66]. Thus, in vivo studies are vital in demonstrating the efficacy and further determining any detrimental effects or toxicity with the long-term usage of BM-derived EVs for therapeutic use. This will eventually aid in deploying BM-derived EVs for various therapeutic applications.

## 5. Conclusions

Taken together, this study details the isolation and biophysical characterization of EVs from the milk of four different species of the bovidae family. Furthermore, in this study, for the first time, a comparative proteomic analysis of EVs from cow, buffalo, goat and sheep milk was performed. These results provide several valuable insights into the nature of the cargo contained within the EVs in the milk of these species and their potential role in regulating consumer’s pathophysiology.

## Figures and Tables

**Table 1:** List of top 20 most abundant proteins identified in CoM, BM, GM and SM.

Top 20 Most Abundant Proteins Identified			
CoM	BM	GM	SM
BTN1A1	BTN1A1	BTN1A1	BTN1A1
XDH	XDH	XDH	XDH
MFGE8	CD36	FASN	PAEP
CD36	MFGE8	CSN1S1	CSN1S1
ABCG2	CSN1S1	PLIN2	LOC101115115
FASN	CSN3	ABCG2	ABCG2
SLC34A2	FABP3	ACTG1	ALB
ALB	ALB	CSN3	PLIN2
FABP3	RAB1A	ACTB	EZR
EZR	PLIN2	ALB	MFGE8
PLIN2	ABCG2	CSN1S2	HSPA8
ENPP3	SLC34A2	EZR	ACTB
RAB1A	FASN	MFGE8	ACTG1
IDH1	RAB1B	PAEP	STOM
HSPA8	RAB18	STOM	CD36
RAB18	EZR	CD36	MSN
MSN	LOC102406615	MSN	RAB18
ACTB	PAEP	ACTC1	CSN2
ACTG1	ENPP3	ACTA1	FASN
PAEP	MSN	ACTG2	FABP3

## Data Availability

Proteomics data has been attached in Supplementary Information (Table S1).

## Supplementary Materials

The following supporting information can be downloaded at: https://www.mdpi.com/article/10.3390/cells12202491/s1, Figure S1. Representative figure for the Gram staining of milk from four species. The milk samples were predominantly enriched with Gram-positive bacilli and cocci. The figures are representative of results from staining three biological replicates. Scale bars represent 200 µm. Figure S2. Abundant proteins in EVs sourced from various species are highly conserved. Venn diagram comparing top 20 most abundant proteins identified in EVs from CoM, BM, GM, and SM. In total, 11 of the top 20 abundant proteins were conserved among the four species. Figure S3. Functional enrichment analysis using FunRich was performed for EVs from all four samples. Histograms representing top six biological pathways for proteins exclusively present in (a) CoM and (b) GM samples. Figure S4. BM EVs induce a higher percentage of cell death. An FACS-based cell death assay was performed on LIM1215 cells by treating them with MEVs from CoM, BM, GM and SM samples (100 µg/mL) for 72 h. (a) Cell cycle distribution pattern for LIM1215 cells when treated with MEVs from CoM, BM, GM and SM samples (100 µg/mL) compared to untreated (UT) control. (b) Percentage of cell death is represented as percent sub-G1 along with other phases of cell cycle (G1/G0, S and G2) which do not show any significant change. (All data are represented as mean ± SEM. * *p* < 0.05, ** *p* < 0.01, *** *p* < 0.001, **** *p* < 0.0001 as determined with an unpaired two-tailed *t*-test). Table S1. Lists of proteins identified in cow, buffalo, sheep and goat milk EVs.
