# Supplementary figures and images for: Isolation and Characterization of Cow-, Buffalo-, Sheep- and Goat-Milk-Derived Extracellular Vesicles
Source: Cells. 2023 Oct 20;12(20):2491. doi: 10.3390/cells12202491 (PMC10605021; doi:10.3390/cells12202491)

Figure S1

**CoM**

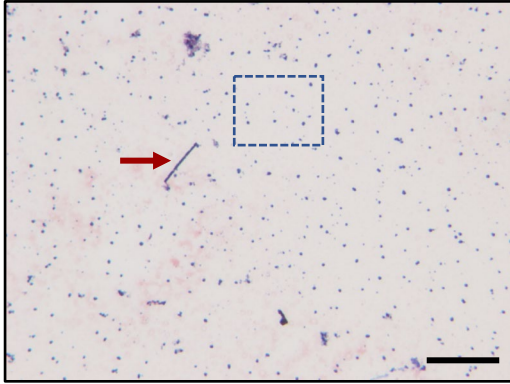

**BM**

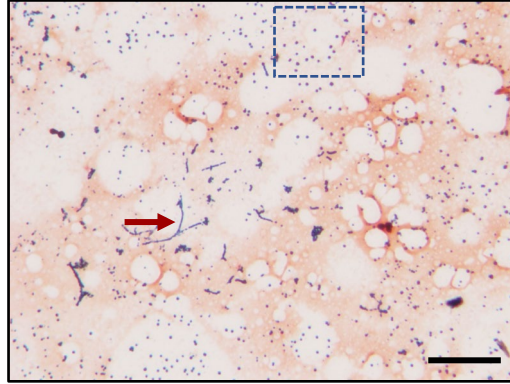

**GM**

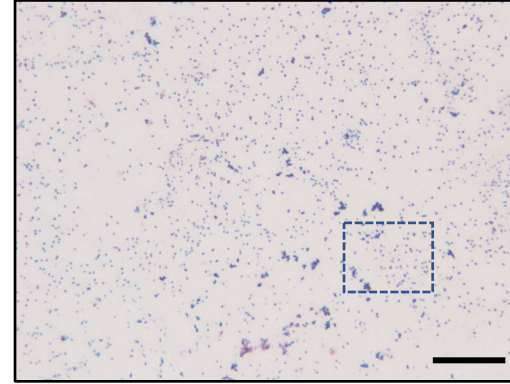

**SM**

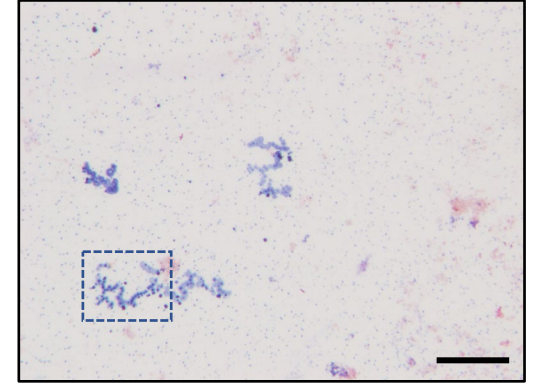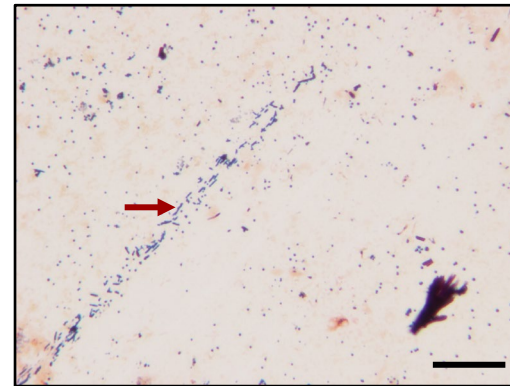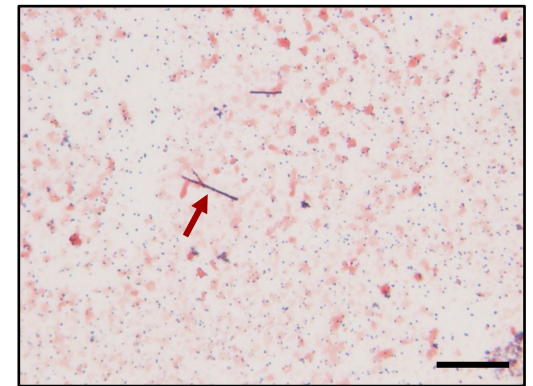

Figure S2

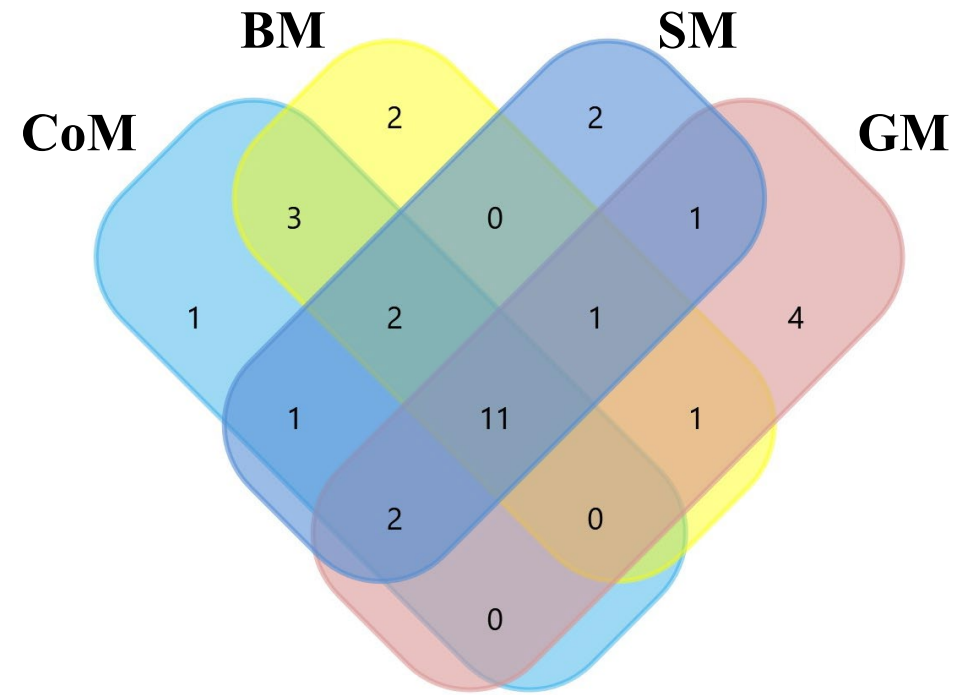

Figure S3

a

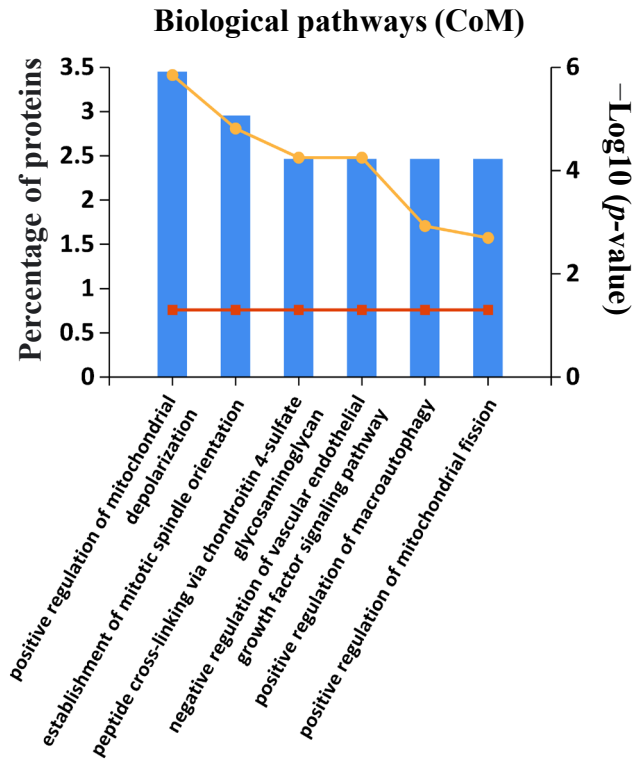

b

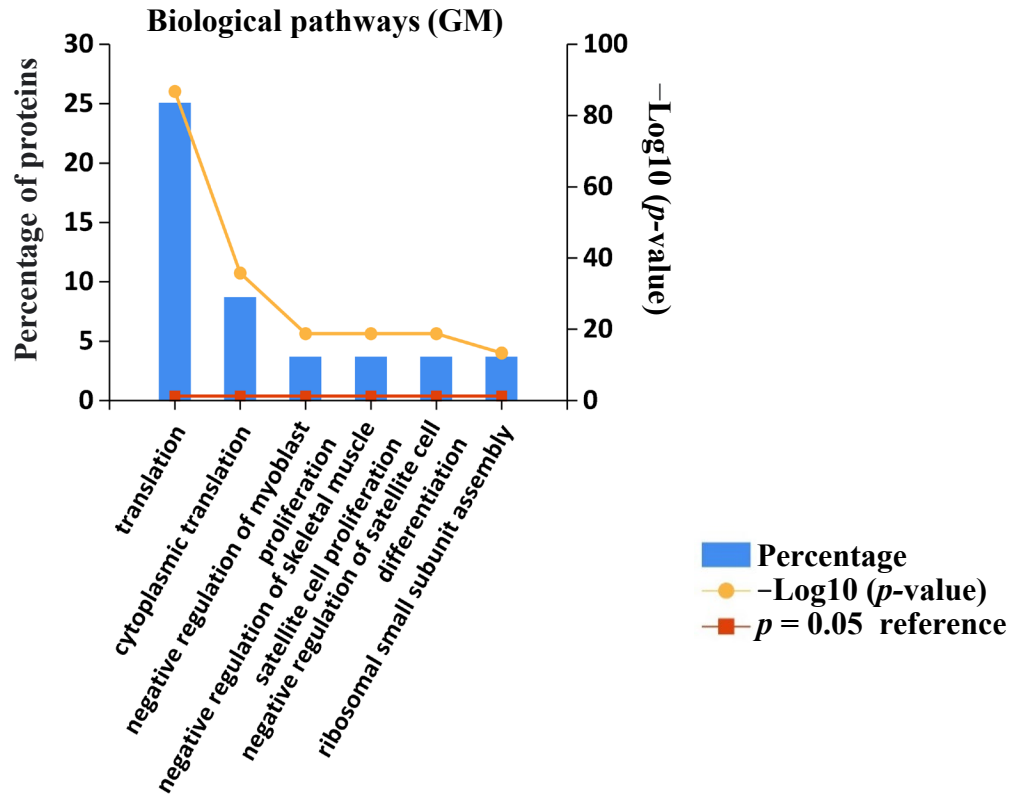

Figure S4

a

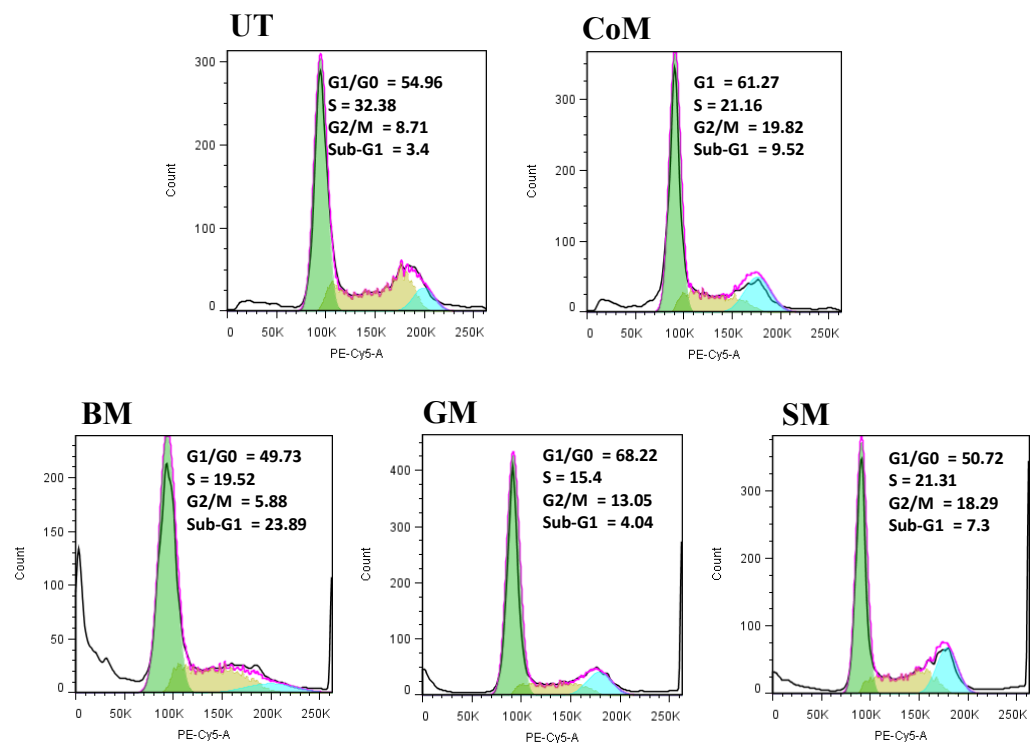

b

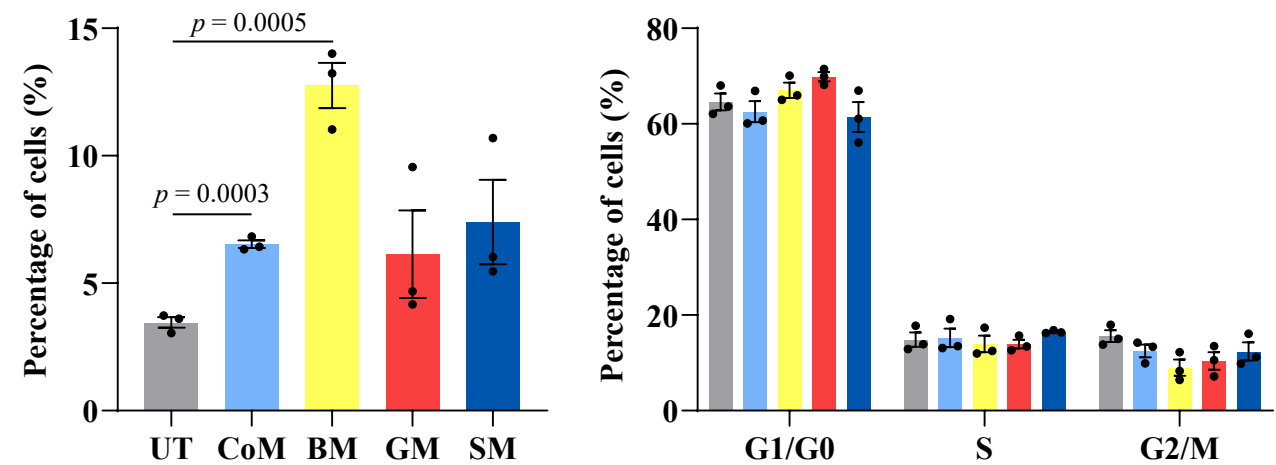

Supplement: Supplementary file 1 [file cells-12-02491-s001.zip › Supplementary figures.pdf]
